# Simultaneous Determination of Ferulic Acid and Vanillin in Vanilla Extracts Using Voltammetric Sensor Based on Electropolymerized Bromocresol Purple

**DOI:** 10.3390/s22010288

**Published:** 2021-12-31

**Authors:** Guzel Ziyatdinova, Anastasiya Zhupanova, Rustam Davletshin

**Affiliations:** 1 Department of Analytical Chemistry, Kazan Federal University, Kremleyevskaya, 18, 420008 Kazan, Russia; Zhupanova.Nastya@mail.ru; 2 Department of High Molecular and Organoelement Compounds, Kazan Federal University, Kremleyevskaya, 18, 420008 Kazan, Russia; RustRDavletshin@kpfu.ru

**Keywords:** electropolymerization, triphenylmethane dyes, voltammetry, natural phenolic antioxidants, food analysis

## Abstract

Natural phenolic antioxidants are one of the widely studied compounds in life sciences due to their important role in oxidative stress prevention and repair. The structural similarity of these antioxidants and their simultaneous presence in the plant samples stipulate the development of methods for their quantification. The current work deals with the simultaneous determination of vanillin and its bioprecursor ferulic acid using a voltammetric sensor for the first time. A sensor based on the layer-by-layer deposition of the polyaminobenzene sulfonic acid functionalized single-walled carbon nanotubes (f-SWCNTs) and electropolymerized bromocresol purple has been developed for this purpose. The best response of co-existing target analytes was registered for the polymer obtained from the 25 µM dye by 10-fold potential cycling from 0.0 to 1.2 V with the scan rate of 100 mV s^−1^ in 0.1 M phosphate buffer (PB), pH 7.0. Scanning electron microscopy (SEM), cyclic voltammetry and electrochemical impedance spectroscopy (EIS) confirmed the effectivity of the sensor developed. The linear dynamic ranges of 0.10–5.0 µM and 5.0–25 µM for both analytes with the detection limits of 72 nM and 64 nM for ferulic acid and vanillin, respectively, were achieved in differential pulse mode. The sensor was applied for the analysis of vanilla extracts.

## 1. Introduction

Natural phenolic antioxidants are one of the widely studied compounds in life sciences due to their important role in oxidative stress prevention and repair [1,2], as well as active application in the food industry [3,4]. Structural similarity of this group of antioxidants and simultaneous presence in the plant samples stipulate the development of methods for their quantification. Separation methods such as chromatography and electrophoresis are traditionally used for the simultaneous determination of natural phenolic antioxidants in the samples of plant origin or foodstuff [5,6,7,8,9,10]. Being electroactive compounds, natural phenolics can be determined by electrochemical methods, including the application of various types of voltammetric sensors based on different modifiers [11,12]. Nevertheless, the majority of them is characterized by insufficient selectivity towards the target analyte in the presence of other structurally similar phenolic antioxidants. Therefore, the development of novel voltammetric sensors for the simultaneous quantification of natural phenolic antioxidants is of high importance.

Among a wide range of electrode surface modifiers, electropolymerized films are of interest due to the easiness of preparation and good retention at the electrode surface, as well as high selectivity and sensitivity to different analytes, including natural phenolic antioxidants [13,14,15]. The electropolymerization of dyes of different classes has been successfully used in electrochemical sensor fabrication. Both electroactive and inert films are reported depending on monomer structure and conditions of electropolymerization. Thus, highly selective voltammetric sensors based on the electropolymerized dyes have been developed for the simultaneous determination of phenolic acids [16,17] and flavonoids [18,19] in foodstuff and medicinal plants. Triphenylmethane dyes containing phenolic fragments in their structure can be considered as promising ones for the preparation of polymeric coverage [17,18,19]. The presence of phenolic fragments in the polymer structure provides sensor response towards structurally related natural phenolic antioxidants in simultaneous presence, as shown by the example of gallic and ellagic acids [17], hesperidin and naringin [18] and rutin and quercetin [19]. 

**Figure 1:**
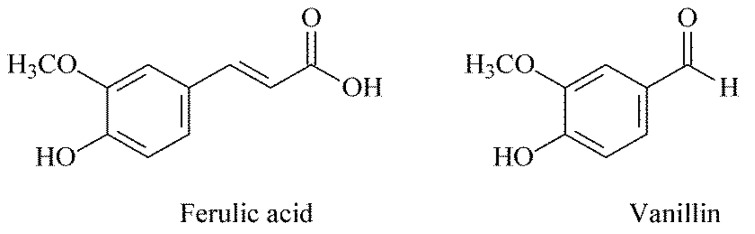
Structures of ferulic acid and vanillin.

Another way of further development in this topic is the quantification of co-existing ferulic acid and vanillin (Figure 1), being of interest for plant material control, including food products. Ferulic acid is a biological precursor of vanillin in *Vanilla planifolia*. The bioconversion can proceed by various pathways depending on the type of microorganisms or enzyme and the plant source [20,21].

To date, the simultaneous determination of ferulic acid and vanillin has been performed using high-performance liquid [22,23] and high-performance thin-layer chromatography [24]. Although both compounds are oxidized electrochemically, there is no information regarding their simultaneous determination. It is probably explained by the structural identity of the electroactive fragments. 

The aim of the current work is the creation of a voltammetric sensor for the simultaneous determination of ferulic acid and vanillin based on the layer-by-layer combination of the polyaminobenzene sulfonic acid functionalized single-walled carbon nanotubes (f-SWCNTs) and electropolymerized bromocresol purple. The conditions of potentiodynamic electropolymerization were optimized. The electrooxidation of ferulic acid and vanillin was studied and the corresponding parameters were calculated. The analytical capabilities of the sensor developed in vanilla extract analysis were evaluated.

## 2. Materials and Methods

### 2.1. Reagents

Ten millimolar stock solutions were prepared in ethanol (rectificate) from ferulic acid (99% purity) and 99% vanillin from Aldrich (Darmstadt, Germany). Bromocresol purple of 90% purity from Sigma-Aldrich (Germany) was used. The selectivity test was performed using 99% ascorbic and gallic acids from Sigma (Germany), 97% vanillic acid from Fluka (Siegen, Germany), with which 10 mM stock solutions were prepared in 5.0 mL of ethanol. The exact dilution was used for the preparation of lower concentrations of the studied compounds.

The homogeneous suspension of f-SWCNTs of 1.0 mg mL^−1^ (*d* × *l* of 1.1 nm × 0.5–1.0 μm; Sigma-Aldrich, Germany) in dimethylformamide was obtained by treatment in an ultrasonic bath (WiseClean WUC-A03H; DAIHAN Scientific Co., Ltd., Wonju-si, Republic of Korea) for 30 min.

Other chemicals were of chemically pure grade and used as received. The laboratory temperature (25 ± 2 °C) was used in the investigations.

### 2.2. Apparatus

A potentiostat/galvanostat PGSTAT 302N with FRA 32M module (Eco Chemie B.V., Utrecht, The Netherlands) and with NOVA 1.10.1.9 software, a 10 mL glass electrochemical cell, a glassy carbon electrode (GCE) with a 3 mm diameter (CH Instruments, Inc., Bee Cave, TX, USA) or modified GCE, a Ag/AgCl/saturated KCl electrode and a platinum auxiliary electrode were used for the electrochemical measurements.

The supporting electrolyte pH was evaluated using the “Expert-001” pH meter (Econix-Expert Ltd., Moscow, Russian Federation) with the glass electrode.

A high-resolution field emission scanning electron microscope Merlin^TM^ (Carl Zeiss, Oberkochen, Germany), operated at the accelerating voltage of 5 kV and the emission current of 300 pA, was used for the characterization of the scanning electron microscopy (SEM) images of the electrodes.

### 2.3. Procedures

#### 2.3.1. Sensor Preparation

The GCE surface was mechanically polished using aluminum oxide powder (with a 0.05 µm grain size) and cleaned in series with acetone and double-distilled water. Then, 2 μL of f-SWCNT suspension was drop-casted onto the GCE surface.

The electrodeposition of poly(bromocresol purple) (polyBCP) was performed in potentiodynamic mode. The electropolymerization conditions (supporting electrolyte pH, concentration of the monomer, number of cycles, electrochemical window and potential scan rate) were optimized based on the voltammetric response of ferulic acid and vanillin mixture.

#### 2.3.2. Electrochemical Measurements

Britton–Robinson buffer (BRB) of pH 2.0–11.0 was used as a supporting electrolyte. The voltammetric measurements were performed after five scans of supporting electrolyte. Cyclic voltammograms (CVs) were recorded from 0.4 to 1.2 V at the potential scan rate of 100 mV s^−1^. Differential pulse voltammograms (DPVs) were registered from 0.5 to 1.2 V. The modulation parameters were varied. DPVs were presented after baseline correction in NOVA 1.10.1.9.

Electrochemical impedance spectroscopy (EIS) was performed in 0.1 M KCl using 1.0 mM [Fe(CN)_6_]^4−/3−^ ions as a redox probe. The potential frequency from 10 kHz to 0.04 Hz was used, the amplitude of sine potential was equal to 5 mV and the equilibrium potential of 0.26 V was calculated as a half sum of 1.0 mM [Fe(CN)_6_]^4−/3−^ ion peak potentials on CVs recorded on the modified electrode. Randles’ equivalent circuit, consisting of *R*_s_ (electrolyte resistance), *Q* (constant phase element), *R*_et_ (electron transfer resistance) and *W* (Warburg element), was applied for the EIS spectra fitting [25]. The χ^2^ parameter was used for the fitting error characterization.

#### 2.3.3. Vanilla Planifolia Extracts Analysis

Commercial extracts of *Vanilla planifolia* (two-fold and three-fold strength) were studied. After filtration and 5-fold dilution of the extract, 20 μL were inserted in the electrochemical cell containing 4.98 mL of BRB, pH 2.0, and DPVs were recorded in the range 0.5–1.2 V at modulation amplitude 75 mV and modulation time of 25 ms, at the scan rate of 10 mV s^−1^.

#### 2.3.4. Statistical Analysis

The statistical analysis of the data obtained was performed at *p* = 0.95 for five parallel measurements (three measurements for chromatography). The average value and coverage interval were used for the presentation of the experimental data. The relative standard deviation was used for the random error characterization. Validation of the developed voltammetric sensor was performed using *t*- and *F*-tests.

OriginPro 8.0 (OriginLab, Northampton, MA, USA) was applied for the regression analyses.

## 3. Results and Discussion

### 3.1. Electropolymerization of Bromocresol Purple and Its Optimization

**Figure 2:**
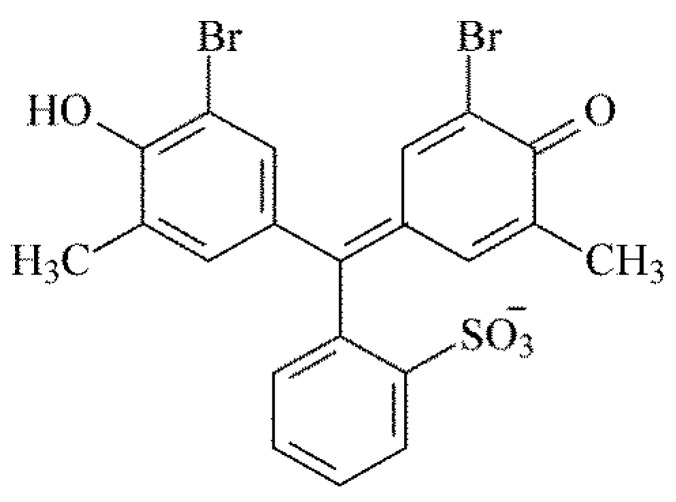
Bromocresol purple structure at pH 7.0.

Bromocresol purple (Figure 2) is a triphenylmethane dye, electrochemically active due to the presence of phenolic fragments. As known [17,18,19], phenol-containing triphenylmethane dyes form non-conductive polymeric coverage. Therefore, the electrodeposition of polyBCP was performed on the f-SWCNTs/GCE. In this case, the high surface area due to the f-SWCNTs provided high loading of the polymeric film and its more uniform distribution on the surface of the electrode. Furthermore, the sufficient conductivity of the electrode was achieved due to the presence of f-SWCNTs preventing blockage of the electron transfer.

**Figure 3:**
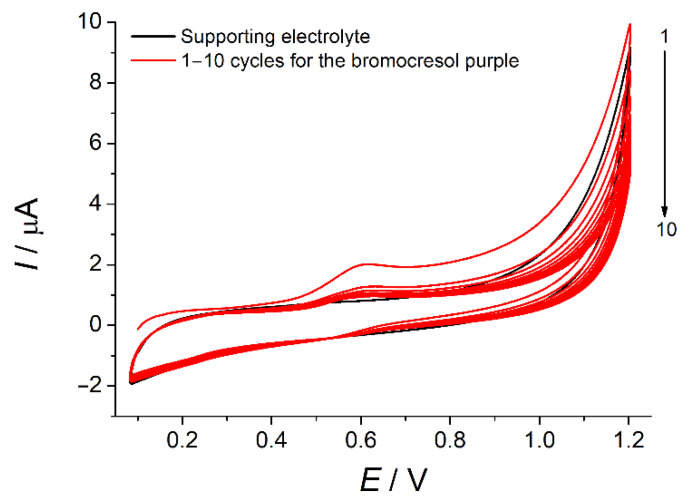
CVs of 25 µM bromocresol purple on the f-SWCNTs/GCE. The supporting electrolyte was 0.1 M phosphate buffer, pH 7.0, υ = 100 mV s^–1^.

There was a well-defined irreversible oxidation peak at 0.60 V on the CVs of bromocresol purple on the f-SWCNTs/GCE in 0.1 M phosphate buffer, pH 7.0 (Figure 3, curve 1). Further potential cycling showed a significant decrease in the oxidation currents (Figure 3, curves 2–9), that is typical for phenolic compounds and confirms the formation of the non-conductive polymer [13,17,18,19]. These data agree well with those reported in [26]. The oxidation currents became stable after the eighth cycle, implying a self-adjustment of the polymer film thickness.

**Scheme 1:**
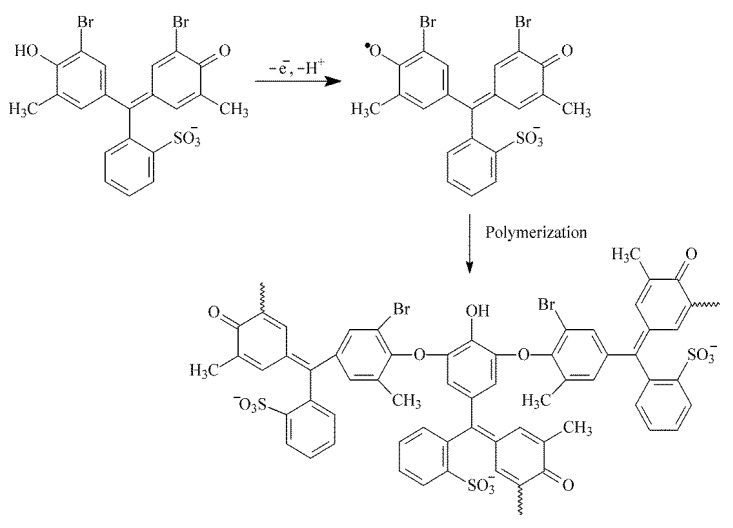
Electropolymerization of bromocresol purple.

Similar to bromophenol blue [27], the oxidation of bromocresol purple proceeds via the formation of phenoxyl radicals that undergo further reactions of dimerization and polymerization, according to Scheme 1.

The electropolymerization conditions affect the properties of final coverage and, consequently, the response of the electrode fabricated towards target analytes. Taking into account the data for the electropolymerization of structurally related thymolphthalein [19], the phosphate buffer at pH 7.0 was chosen as a supporting electrolyte. The investigations in the basic medium showed a significant decrease in the bromocresol purple oxidation currents due to its chemical oxidation by air oxygen. The anodic shift of bromocresol purple oxidation potential is observed in acidic media that hinder the electron detachment, as well as significantly narrowing the available electrochemical window. 

**Figure 4:**
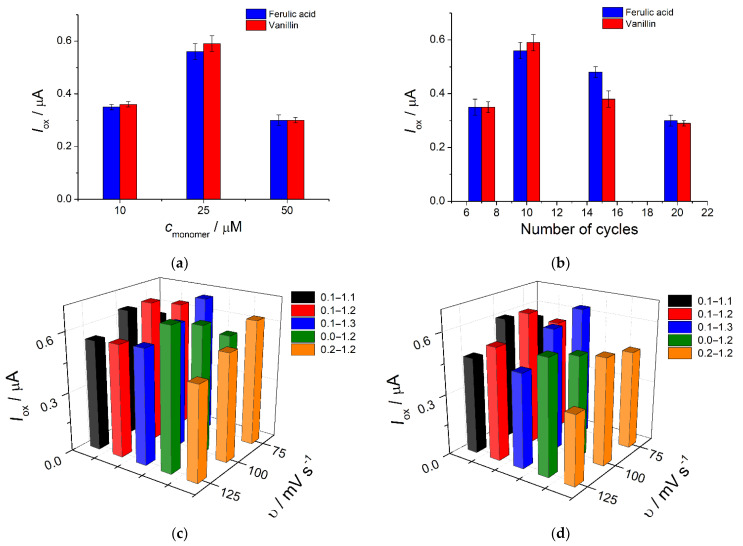
Effect of bromocresol purple electropolymerization conditions on the oxidation currents of 10 µM mixture of ferulic acid and vanillin. (**a**) Effect of monomer concentration: polarization window, 0.0–1.0 V; υ = 100 mV s^−1^. (**b**) Effect of number of cycles: polarization window, 0.0–1.0 V; υ = 100 mV s^−1^; *c*_monomer_ = 25 µM. (**c**) Effect of polarization window and potential scan rates on the oxidation currents of ferulic acid: number of cycles, 10; *c*_monomer_ = 25 µM. (**d**) Effect of polarization window and potential scan rates on the oxidation currents of vanillin: number of cycles, 10; *c*_monomer_ = 25 µM.

Other conditions of bromocresol purple electropolymerization were optimized using voltammetric characteristics of the analytes, i.e., ferulic acid and vanillin mixture, under conditions of cyclic voltammetry in BRB, pH 2.0. The peak potential separation of ferulic acid and vanillin was not affected by the conditions of electropolymerization and equaled 170 ± 5 mV. Therefore, the oxidation currents of the 10 µM mixture of ferulic acid and vanillin were considered. The variation in dye concentration in the range of 10–50 µM (Figure 4a) showed that the best response was obtained for 25 µM of bromocresol purple. The number of cycles affected the thickness of the polymeric film and indicated that ten cycles were enough to obtain maximal oxidation currents of ferulic acid and vanillin (Figure 4b). A further increase in the cycle number led to the statistically significant decrease in the response of both analytes due to the growth of non-conducting polymeric coverage thickness, leading to the blockage of active centers on the electrode surface. As one can see from Figure 4c,d, the electrolysis parameters significantly affected the oxidation currents of ferulic acid and vanillin. There was no clear trend of the changes observed, although the decrease in the analytes’ oxidation currents for the potential scan rate of 125 mV s^−1^ was registered for all polarization windows, excluding 0.0–1.2 V. This was probably caused by the effect of the start potential and electrolysis time. The best response of both ferulic acid and vanillin was obtained in the case of the polarization window from 0.1 to 1.2 V and potential scan rate of 100 mV s^−1^. Thus, the electropolymerization of bromocresol purple had to be performed from its 25 µM solution in 0.1 M phosphate buffer, pH 7.0, by 10-fold potential cycling from 0.1 to 1.2 V with the scan rate of 100 mV s^−1^.

### 3.2. Characterization of the Electrodes

#### 3.2.1. Electrode Surface Morphology

**Figure 5:**
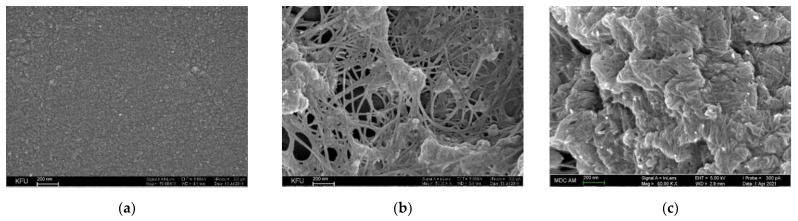
SEM images of bare GCE (**a**), f-SWCNTs/GCE (**b**) and polyBCP/f-SWCNTs/GCE (**c**).

The surface of the bare GCE and those of the modified electrodes were studied by SEM (Figure 5).

GCE had typical morphology of low roughness (Figure 5a). f-SWCNTs formed a strongly interwoven mesh structure with an average thickness of individual nanotubes of 21–28 nm (Figure 5b). The polyBCP layer consisted of folded structures of high roughness with channels (Figure 5c) that were fairly evenly distributed on the electrode surface. The SEM data confirmed the changes in the electrodes surface morphology and the immobilization of the modifiers. The high roughness of the electrodes surface led to the increase in their working area.

#### 3.2.2. Effective Surface Area of the Electrodes

**Figure 6:**
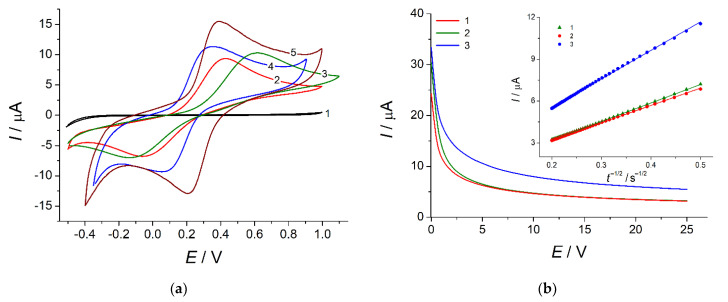
(**a**) CVs of 1.0 mM [Fe(CN)_6_]^4−^ on bare GCE (curve 2), polyBCP/GCE (curve 3), f-SWCNTs/GCE (curve 4) and polyBCP/f-SWCNTs/GCE (curve 5) in 0.1 M KCl (curve 1), υ = 100 mV s^−^^1^. (**b**) Chronoamperograms of 1.0 mM [Fe(CN)_6_]^4−^ on bare GCE (curve 1), polyBCP/GCE (curve 2) and f-SWCNTs/GCE (curve 3). Insert is the plot of *I* vs. *t* ^−1/2^ obtained from chronoamperometric measurements.

The effective surface of the electrodes under consideration was evaluated using the electrochemical approach. One millimolar hexacyanoferrate(II) ions were used as a standard redox probe. Their oxidation in 0.1 M KCl was studied (Figure 6a). The quasi-reversible electrooxidation of hexacyanoferrate(II) ions was observed on the bare GCE and f-SWCNTs/GCE as the cathodic-to-anodic peak potential separation was confirmed (Figure 6a, curves 2 and 4). The decrease in the hexacyanoferrate(II) ions oxidation reversibility was observed on the polyBCP-modified GCE (Figure 6a, curve 3), which is explained by the low conductivity of the polymeric coverage. PolyBCP/f-SWCNTs/GCE exhibited the improvement of the hexacyanoferrate(II) ions oxidation reversibility and a significant increase in the redox currents (Figure 6a, curve 5). These data agree well with those reported for polyaluminon [18] and polythymolphthalein [19].

Chronoamperometry and the Cottrell equation [28] were applied for the estimation of the electroactive area of the bare GCE, polyBCP/GCE and f-SWCNTs/GCE on the basis of *I* vs. *t* ^−1/2^ plots (Figure 6b, insert). Cyclic voltammetry data and the Randles–Ševčík equation [28] were used for the polyBCP/f-SWCNTs/GCE. The electrodeposition of polyBCP on the surface of the bare GCE led to insignificant changes in the effective surface area (8.25 ± 0.5 mm^2^ vs. 8.2 ± 0.1 mm^2^ for the bare GCE) that agree well with the cyclic voltammetry data. A statistically significant increase in the effective surface area was obtained for f-SWCNTs/GCE and polyBCP/f-SWCNTs/GCE (13.6 ± 0.2 and 42 ± 1 mm^2^, respectively) that agrees well with the SEM results. Moreover, the application of f-SWCNTs as a platform for polymer deposition had a strong effect on the electrode effective surface area and other parameters, as was shown in further experiments.

#### 3.2.3. Electron Transfer Properties

**Figure 7:**
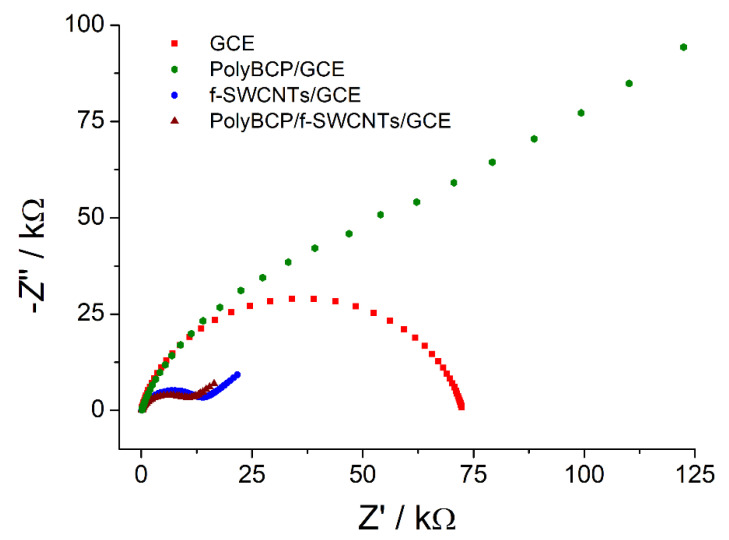
Nyquist plots for bare GCE and modified electrodes in the presence of 1.0 mM [Fe(CN)_6_]^4^^−/3^^−^ in 0.1 M KCl. *E* = 0.26 V; frequency range, 10 kHz—0.04 Hz (12 frequency steps per decade); amplitude, 5 mV.

EIS, using a 1.0 mM hexacyanoferrate(II)/(III) mixture as a redox probe, was applied for the characterization of the electron transfer properties of the electrodes. The polarization potential of 0.26 V, calculated as a half-sum of the probe redox potentials, was used. The corresponding Nyquist plots are presented in Figure 7. Significant changes in the electron transfer resistance reflected by semicircle diameter in the high frequencies range were observed for modified electrodes in comparison to the bare GCE. The polyBCP-based GCE showed an increase in the electron transfer resistance in comparison to the bare GCE that was caused by the non-conducting properties of polymeric coverage. A similar behavior has been reported for electropolymerized natural phenolics [13,29,30]. Electrodes with a f-SWCNT layer demonstrated significantly lower electron transfer resistance than the bare GCE, that agrees well with data for other electropolymerized triphenylmethane dyes [18,19]. PolyBCP/f-SWCNTs/GCE was characterized by the lowest electron transfer resistance among the electrodes under consideration, which is in line with the data of cyclic voltammetry for [Fe(CN)_6_]^4−^ ions.

The quantitative characteristics of the impedance spectrum fitting are presented in Table 1. The changes in the electron transfer resistance allowed us to conclude that the polyBCP/f-SWCNT-based electrode demonstrated a 7.2-fold higher electron transfer rate than the bare GCE. The increase in the electrode surface roughness was also confirmed by the constant phase element and *n* factor values. The χ^2^ values indicated high accuracy of the fitting with the equivalent circuits used.

Thus, SEM and electrochemical method data showed the effectivity of the application of polyBCP/f-SWCNTs as electrode surface modifier.

### 3.3. Cyclic Voltammetry of Ferulic Acid and Vanillin on Bare and Modified Electrodes

**Figure 8:**
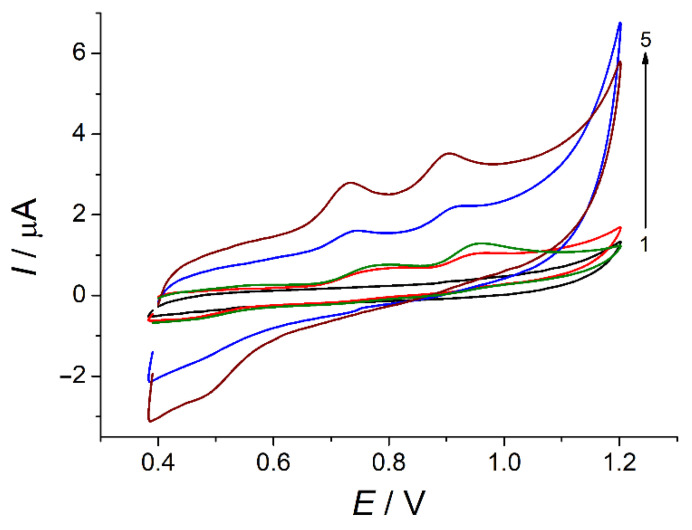
CVs of 10 µM mixture of ferulic acid and vanillin on bare GCE (curve 2), polyBCP/GCE (curve 3), f-SWCNTs/GCE (curve 4) and polyBCP/f-SWCNTs/GCE (curve 5) in BRB, pH 2.0 (curve 1), υ = 100 mV s^−1^.

The voltammetric behavior of 10 µM ferulic acid and vanillin mixture in BRB, pH 2.0, was studied. There were two irreversible peaks, at 0.773 V and 0.944 V, on the CVs of the bare GCE corresponding to the oxidation of ferulic acid and vanillin, respectively (Figure 8). The oxidation currents of 0.15–0.17 µA indicate insufficient sensitivity of the electrode response. Moreover, the oxidation peaks almost disappeared at concentrations lower than 5.0 µM. 

PolyBCP/f-SWCNTs/GCE was used to enhance the voltammetric characteristics of ferulic acid and vanillin. As one can see from Figure 8, curve 5, a significant improvement of the shape of the voltammograms, as well as an increase in both ferulic acid and vanillin oxidation currents (4.12- and 3.82-fold, respectively, vs. the bare GCE), was registered on the polymer-based electrode. 

To elucidate the effect of modifiers, the voltammetric characteristics of ferulic acid and vanillin on the f-SWCNTs/GCE and polyBCP/GCE were tested. The GCE modified with polyBCP showed 1.35- and 2.52-fold higher oxidation currents of ferulic acid and vanillin, respectively, than the bare GCE (Figure 8, curve 3). The oxidation potential of ferulic acid remained unchanged, while a 13 mV anodic shift was observed for vanillin. These data confirm that the polymer’s and analytes’ nature had a significant impact on the voltammetric response obtained. The modification of the electrode surface with f-SWCNTs provided a 1.65- and 1.35-fold increase in the oxidation currents and cathodic shifts of the oxidation potentials of 40 mV and 30 mV for ferulic acid and vanillin, respectively, indicating the electrocatalytic effect of f-SWCNTs. Thus, the synergetic effect of both co-modifiers was confirmed. Furthermore, higher oxidation currents of ferulic acid and vanillin on the f-SWCNTs/GCE and polyBCP/f-SWCNTs/GCE were caused by the increase in the electroactive surface area of the modified electrodes vs. the bare GCE. The electrode based on the combination of f-SWCNTs and polyBCP could be used for the simultaneous detection of ferulic acid and vanillin with satisfactory sensitivity.

### 3.4. Electrooxidation of Ferulic Acid and Vanillin on Polymer-Based Electrode

The electrooxidation parameters of ferulic acid and vanillin were evaluated based on the study of supporting electrolyte pH and potential scan rate effect on the voltammetric characteristics.

**Figure 9:**
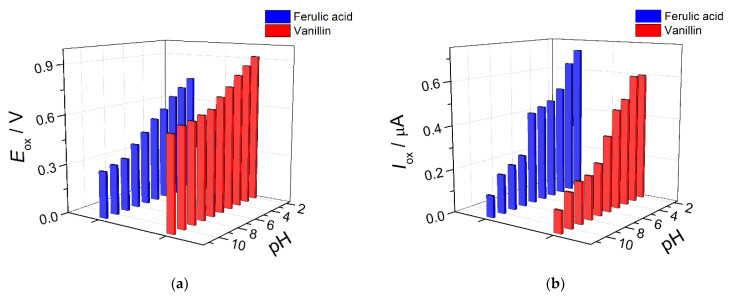
Effect of supporting electrolyte pH on the oxidation potentials (**a**) and currents (**b**) of 10 µM ferulic acid and vanillin on polyBCP/f-SWCNTs/GCE; υ = 100 mV s^−1^.

BRB’s pH was varied in the range 2.0–11.0. The one-step irreversible oxidation was observed for both analytes in the whole pH range under consideration. The oxidation potentials of both ferulic acid and vanillin were shifted to the less positive values with the pH’s increase (Figure 9a), which means the participation of the protons in the electrooxidation. The oxidation currents were statistically significantly decreased with the pH’s growth (Figure 9b), that is typical for phenolic compounds due to the partial chemical oxidation by air oxygen that is intensified in a basic medium [11]. The best peak potential separation and the highest oxidation currents of ferulic acid and vanillin were registered at pH 2.0, which was used in further investigations.

**Figure 10:**
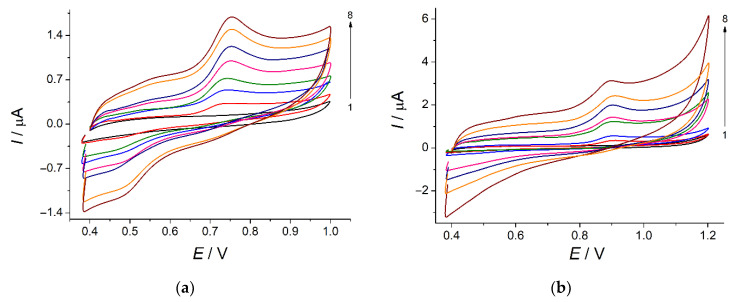
CVs of 10 µM ferulic acid (**a**) and vanillin (**b**) on polyBCP/f-SWCNTs/GCE in BRB, pH 2.0 (curve 1), at various potential scan rates (10 (curve 2), 25 (curve 3), 50 (curve 4), 75 (curve 5), 100 (curve 6), 125 (curve 7) and 150 (curve 8) mV s^−1^).

The changes in the oxidation potentials and currents of ferulic acid and vanillin at the various potential scan rates were studied in order to evaluate the electrooxidation parameters (Figure 10).

The oxidation currents of both analytes were linearly increased as a function of the square root of the potential scan rate (Table 2). Furthermore, the Napierian logarithmic dependence of oxidation currents vs. potential scan rate had a slope of 0.66 and 0.58 for ferulic acid and vanillin, respectively. Thus, electrooxidation of both phenolics was a diffusion-driven process, which agrees with data for poly(methyl orange)-modified graphene paste electrode [31] and poly(1H-1,2,4-triazole-3-thiol)-based gold electrode [32] for vanillin, and poly(sunset yellow)-based sensor [16] and multi-walled carbon nanotubes-modified glassy carbon electrode [33] for ferulic acid.

There was no cathodic peak on the cyclic voltammograms for vanillin. A weakly pronounced reduction peak around 0.5 V was observed for ferulic acid. The cathodic-to-anodic peak potential separation was more than 200 mV. Therefore, the electrooxidation of both ferulic acid and vanillin proceeded irreversibly. According to the Tafel plots at the low-potential scan rates [28], the anodic transfer coefficients of 0.52 and 0.47 were calculated for ferulic acid and vanillin, respectively. The number of electrons participating in electrooxidation was obtained from Equation (1) [28] and is equal to 2.2 for ferulic acid and 2.3 for vanillin, which is in line with what is reported in [16,32,33,34,35,36,37,38].

∆*E*_1/2_=47.7/α_a_*n* (1)

**Scheme 2:**
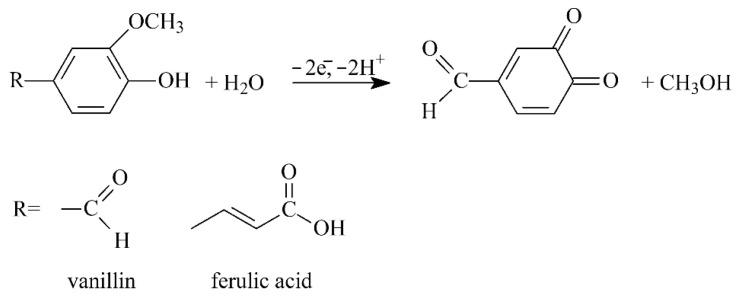
Electrooxidation of ferulic acid and vanillin.

Thus, the electrooxidation of ferulic acid and vanillin led to the formation of *o*-quinones (Scheme 2).

The diffusion coefficients and the standard heterogeneous electron transfer rate constants were calculated using Equations (2) [28] and (3) [39], which are valid for the irreversible diffusion-driven electrooxidation.

(2) Iox=π12χ(bt)nFAcD12(αanαFRT)12υ12

(3) k0=2.415e−0.02FRTD12(Ep−Ep/2)−12υ12

The diffusion coefficients equaled to (1.0 ± 0.1) × 10^−6^ and (8.3 ± 0.3) × 10^−7^ cm^2^ s^−1^ and the standard heterogeneous electron transfer rate constants of 5.6 × 10^−4^ and 5.4 × 10^−4^ cm s^−1^ were obtained for ferulic acid and vanillin, respectively.

### 3.5. Analytical Characterization of Poly(Bromocresol Purple)-Based Sensor

The quantification of ferulic acid and vanillin using a polyBCP-based sensor was performed in differential pulse mode in order to provide a higher sensitivity of the sensor response. The variation in the pulse parameters (amplitude and time) showed that the oxidation potentials of both ferulic acid and vanillin were insignificantly decreased (at 10 mV) with the increase in the pulse amplitude and time (Figure S1a,b). The oxidation currents of the analytes were statistically significantly increased with the growth in the pulse amplitude (Figure S1c,d). On contrary, the increase in pulse time led to the decrease in the oxidation currents. Nevertheless, the shape of the differential pulse voltammograms was not affected by the pulse parameters. The pulse amplitude of 75 mV and time of 25 ms were used for further measurements as far as providing the best response of ferulic acid and vanillin mixture.

**Figure 11:**
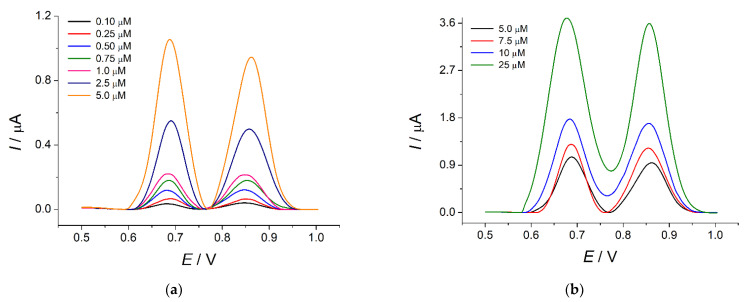

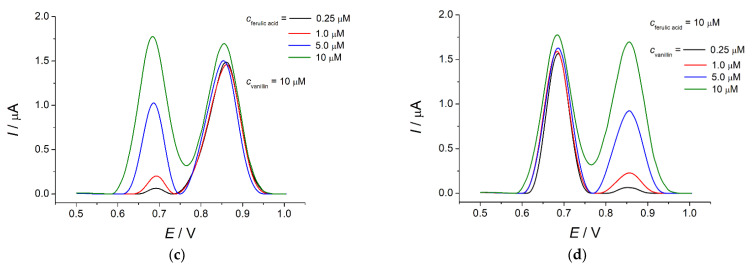
(**a**,**b**) Baseline-corrected DPVs of equimolar mixtures of ferulic acid and vanillin on polyBCP/f-SWCNTs/GCE in BRB, pH 2.0; concentration ranges are 0.10–5.0 µM (**a**) and 5.0–25 µM (**b**). (**c**) Baseline-corrected DPVs of non-equimolar mixtures of ferulic acid and vanillin (various concentrations of ferulic acid at the fixed 10 µM concentration of vanillin). (**d**) Baseline-corrected DPVs of non-equimolar mixtures of ferulic acid and vanillin (various concentrations of vanillin at the fixed 10 µM concentration of ferulic acid). Δ*E*_pulse_ = 75 mV; *t*_pulse_ = 25 ms; υ = 10 mV s^−1^.

There were well-resolved oxidation peaks of ferulic acid and vanillin at 0.69 and 0.86 V, respectively, on the polyBCP-based sensor (Figure 11a,b). The oxidation currents were linearly increased with the increase in both analytes concentration in the ranges 0.10–5.0 µM and 5.0–25 µM (Equations (4) and (5) for ferulic acid and Equations (6) and (7) for vanillin).

*I* [µA] = (0.018 ± 0.005) + (22.8 ± 0.2) × 10^4^ *c* [M] *R*^2^ = 0.9994 (4)

*I* [µA] = (0.43 ± 0.02) + (11.6 ± 0.2) × 10^4^ *c* [M] *R*^2^ = 0.9994 (5)

*I* [µA] = (0.025 ± 0.004) + (18.6 ± 0.2) × 10^4^ *c* [M] *R*^2^ = 0.9995 (6)

*I* [µA] = (0.41 ± 0.01) + (10.8 ± 0.1) × 10^4^ *c* [M] *R*^2^ = 0.9997 (7)

The limits of detection calculated as 3SD_a_/*b*, where SD_a_ is the standard deviation of calibration plot intercept and *b* is the slope of the calibration plot, were 0.072 µM for ferulic acid and 0.064 µM for vanillin. 

The independent electrooxidation of ferulic acid and vanillin was suggested based on the 170 mV oxidation potential difference. The data for non-equimolar mixtures proved this fact (Figure 11c,d) as far as no overlapping peak was observed. Moreover, the oxidation currents of ferulic acid and vanillin, in this case, were similar to their oxidation currents in equimolar mixtures. Thus, the concentration ratio of ferulic acid and vanillin in the mixture did not affect the analytical signal of the polyBCP-based sensor, which could be applied for both individual and simultaneous quantification of the analytes. The calibration plots obtained are universal. The data obtained open wider opportunities for the practical application of the sensor developed.

The analytical characteristics are better than or comparable to those reported to date for the individual determination of ferulic acid or vanillin using electrochemical sensors based on polymeric coverages (Table 3). Other advantages of the polyBCP-based sensor are the simplicity of fabrication and fast response.

The accuracy of the ferulic acid and vanillin simultaneous quantification using the polyBCP-based sensor was studied on their model mixtures (Table 4). The recovery values of 98.7–100% indicate the high accuracy of the sensor response. The absence of the random error in the determination was confirmed by the relative standard deviation values being less than 5%.

#### 3.5.1. Repeatability, Reproducibility and Robustness of Sensor Response

Ferulic acid and vanillin electrooxidation products were adsorbed on the polyBCP-based sensor, leading to a significant decrease in their oxidation currents (up to 30%) when the sensor was used for the second time. Therefore, the sensor surface was fully renewed after each measurement. The excellent reproducibility of the sensor response was confirmed by relative standard deviation values of 4.5% and 4.1% for the 0.10 µM mixture of ferulic acid and vanillin and 1.0% and 0.65% for the 10 µM mixture using five electrodes for each concentration. 

By varying the reference electrode and GCE of the same producer; changing f-SWCNT suspension to a newly prepared one; replacing dimethylformamide, orthophosphoric, boric and acetic acids used for the f-SWCNT suspension; and supporting electrolyte preparation, the robustness of the sensor response was studied. The relative standard deviation of the ferulic acid and vanillin determination was 1.5–3.0%, which confirms the high robustness of the polyBCP-based sensor.

#### 3.5.2. Interference Study

The effect of typical interferences such as inorganic ions and saccharides on the voltammetric response of a 1.0 µM mixture of ferulic acid and vanillin was studied. Thousand-fold excesses of K^+^, Mg^2+^, Ca^2+^, NO_3_^-^, Cl^-^ and SO_4_^2-^ and hundred-fold excesses of glucose, rhamnose and sucrose were electrochemically inactive and did not affect the response of ferulic acid and vanillin. 

Other antioxidants, including structurally related phenolic acids, may affect the sensor response. Therefore, ascorbic, gallic and vanillic acids were considered as potential interferences. Ascorbic acid did not oxidize in the potential window studied. Its 100-fold excess did not lead to changes in the voltammetric characteristics of ferulic acid and vanillin. On the contrary, gallic and vanillic acids were oxidized on the polyBCP/f-SWCNTs/GCE (Figure S2). Gallic acid showed a two-step electrooxidation at 0.50 V and 0.82 V. The first oxidation step of gallic acid did not affect the response of ferulic acid and vanillin. The second step of gallic acid oxidation overlapped with the vanillin’s peak, leading to its proportional increase. Nevertheless, the peak of gallic acid at 0.82 V was poorly expressed and fully disappeared at the 2.5 µM level. Therefore, gallic acid did not interfere at concentrations ≤2.5 µM. Vanillic acid was oxidized at 0.82 V, which affected vanillin detection. The tolerance level of vanillic acid at which the oxidation peak was not observed equaled ≤0.25 µM. In this case, there was no significant change in the oxidation currents of vanillin (±1%) in the mixture.

Thus, the polyBCP-based sensor was characterized by good selectivity towards ferulic acid and vanillin.

### 3.6. Application of the Sensor to Vanilla Planifolia Extract Analysis

The sensor developed was applied in the analysis of commercial *Vanilla planifolia* extracts of two- and three-fold strength that are usually used in the food industry as flavoring additives.

**Figure 12:**
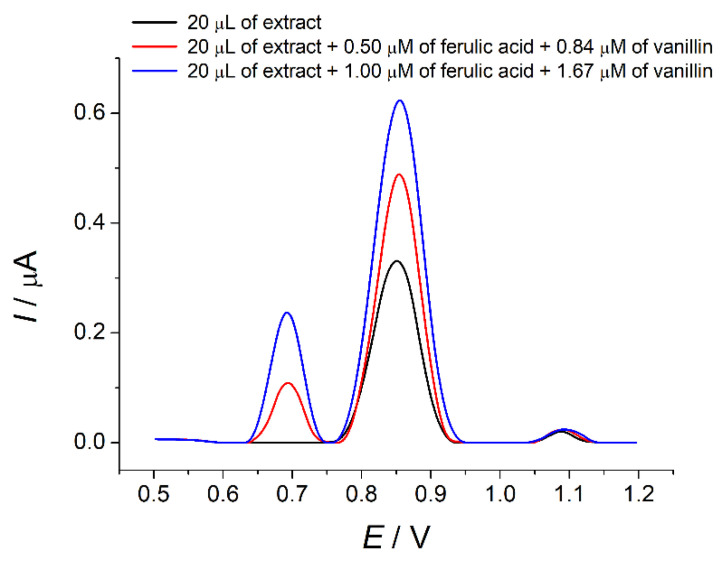
Baseline-corrected DPVs of *Vanilla planifolia* extract in the absence and in the presence of standard additions of ferulic acid and vanillin in the electrochemical cell on polyBCP/f-SWCNTs/GCE in BRB, pH 2.0. Δ*E*_pulse_ = 75 mV; *t*_pulse_ = 25 ms; υ = 10 mV s^−1^.

As Figure 12 shows, the extracts were free of ferulic acid and contained vanillin, which was confirmed by the absence of the oxidation peak of ferulic acid and the well-defined peak of vanillin at 0.85 V. Furthermore, an oxidation peak at 1.09 V of low intensity was observed for two studied samples. The standard addition method (Figure 12) showed a proportional increase in the oxidation currents at 0.69 V and 0.85 V. The third peak was not affected by the addition of ferulic acid and vanillin. The recovery values of 99.6–100% (Table S1) indicate the absence of matrix effects and applicability of polyBCP-based sensor to real sample analysis.

The results of ferulic acid and vanillin quantification in *Vanilla planifolia* extracts are presented in Table 5. The spiked analysis was performed for ferulic acid for the confirmation of the analytical capabilities of the sensor to quantify ferulic acid in the food samples. The analyte was spiked in the diluted extracts prior to measurements.

The electrochemical data were compared to high-performance liquid chromatography with UV detection [23] and a good agreement was obtained. One sample *t*-test indicated the absence of systematic errors of the quantification using the polyBCP-based sensor. *F*-test data were less than the critical value of 6.94 [48], which indicates the similar precision of the electrochemical and chromatographic approaches.

## 4. Conclusions

An electrochemical sensor for the simultaneous quantification of ferulic acid and vanillin was developed for the first time. High sensitivity and selectivity of the sensor response were achieved by the application of a layer-by-layer combination of f-SWCNTs and electropolymerized bromocresol purple. Such approach to the sensor surface modification provided high electroactive area and improvement of the electron transfer rate, as well as sufficient conductivity of the sensor. The conditions of electropolymerization providing the best response of ferulic acid and vanillin were optimized. The electrooxidation parameters of ferulic acid and vanillin were calculated.

The polyBCP-based sensor allowed both individual and simultaneous quantification of target analytes to be performed, as far as the calibration plots obtained are universal. Another advantage of the sensor is improved analytical characteristics compared to other polymer-based electrochemical sensors for the individual determination of ferulic acid or vanillin. The sensor selectivity in the presence of other phenolic acids significantly enlarges its application area.

The poly-BCP-based sensor is simple and express in its preparation, characterized by high reproducibility, robustness and reliability of the response, as well as cost-efficiency, and has a wide application area in the food industry for the control of both flavor additives and final products.

## Figures and Tables

**Table 1:** Electrochemical impedance parameters of the electrodes (*n* = 5; *p* = 0.95).

Electrode	*R*_s_/Ω	*R*_ct_/kΩ	*Q*/µΩ^−1^	*n*	*W*/µΩ^−1^	Χ^2^
GCE	101 ± 6	72.5 ± 0.9	0.45 ± 0.05	0.860	-	0.025
PolyBCP/GCE	105 ± 3	84 ± 1	0.73 ± 0.06	0.822	5.3 ± 0.1	0.027
f-SWCNTs/GCE	109 ± 2	12.4 ± 0.5	2.20 ± 0.05	0.819	152 ± 4	0.033
PolyBCP/f-SWCNTs/GCE	104 ± 1	10.1 ± 0.2	5.4 ± 0.1	0.811	199 ± 1	0.021

**Table 2:** Effect of potential scan rate on the oxidation currents of ferulic acid and vanillin on polyBCP/f-SWCNTs/GCE in BRB, pH 2.0.

Parameter	Ferulic Acid	Vanillin
***I* (µA) = *a* + *b*υ^1/2^ (mV s** **^−1^)**		
*a* ± SD	−0.08 ± 0.01	−0.06 ± 0.02
*b* ± SD	0.058 ± 0.002	0.060 ± 0.002
*R* ^2^	0.9950	0.9901
**ln*I* (µA) = *a* + *b*lnυ (V s^−1^)**		
*a* ± SD	0.80 ± 0.08	0.71 ± 0.08
*b* ± SD	0.66 ± 0.03	0.58 ± 0.03
*R* ^2^	0.9904	0.9886

**Table 3:** Figures of merit for the electrochemical sensors based on the polymeric coverages for the individual determination of ferulic acid and vanillin.

Sensor	Detection Mode	Limit of Detection/µM	Linear Dynamic Range/µM	Refs.
**Ferulic acid**				
Poly(sunset yellow)/Multi-walled carbon nanotubes/GCE	DPV ^1^	0.098	0.5–4.0	[16]
Polypyrrole-multi-walled carbon nanotubes/GCE	AdASWV ^2^	1.17	3.32–25.9	[40]
Molecularly imprinted poly(phenol) film/Au nanoparticles/Reduced graphene oxide/SPE ^3^	DPV	0.0031	0.010–1.0	[41]
**Vanillin**				
Poly(methyl orange)/Graphene paste electrode	DPV	0.0735	10–35 and 40–100	[31]
Poly(1H-1,2,4-triazole-3-thiol)/Gold electrode	DPV	0.04	0.1–11.3	[32]
Poly(3-amino-1,2,4-triazole-5-thiol/ Gold electrode	DPV	0.19	1.1–76.5	[42]
Poly(Alizarin Red S)/GCE	SWV ^4^	0.06	0.5–20 and 20–250	[43]
Polyniacin/Multi-walled carbon nanotubes paste electrode	CV ^5^	1.01	2–35 and 40–80	[44]
Poly(glutamic acid)/Multi-walled carbon nanotubes and graphite paste electrode	DPV	0.0199	0.50–18.0	[45]
Octoxynol-9/Poly(titan yellow)/Carbon nanotubes paste electrode	DPV	0.049	2.0–40	[46]
Molecularly imprinted poly(1-vinyl-3-octylimidazole hexafluoride phosphorus)−multi-walled carbon nanotubes@polydopamine–carboxylated single-walled carbon nanotubes/	DPV	0.1	0.2–10	[47]

^1^ Differential pulse voltammetry. ^2^ Adsorptive anodic square wave voltammetry. ^3^ Screen-printed electrode. ^4^ Squarewave voltammetry. ^5^ Cyclic voltammetry.

**Table 4:** Simultaneous determination of ferulic acid and vanillin in model mixtures (*n* = 5; *p* = 0.95).

Added/µM	Found Ferulic Acid/µM	RSD/%	*R*/%	Found Vanillin/µM	RSD/%	*R*/%
0.10	0.099 ± 0.006	4.7	99.0	0.099 ± 0.005	4.3	99.0
0.75	0.75 ± 0.04	4.6	100	0.75 ± 0.03	3.0	100
2.5	2.50 ± 0.04	1.7	100	2.49 ± 0.05	1.5	99.6
7.5	7.5 ± 0.3	2.7	100	7.48 ± 0.09	0.98	99.7
25	25.0 ± 0.5	0.83	100	25.0 ± 0.2	0.38	100

**Table 5:** Simultaneous quantification of ferulic acid and vanillin in *Vanilla planifolia* extracts (*p* = 0.95).

Sample	Spiked/mg L^−1^	Ferulic Acid			Vanillin					
		Voltammetry/ mg L^−1^	RSD/%	HPLC-UV/ mg L^−^^1^	Voltammetry/ mg L^−1^	RSD/%	HPLC-UV/mg L^−^^1^	RSD/%	*t*-Test ^1^	*F*-Test ^2^
1 (x2)	0	0	-	0	317 ± 6	1.6	321 ± 17	2.1	0.909	1.778
	24.3	24.3 ± 0.6	2.1							
	48.5	48.4 ± 0.7	1.5							
2 (x3)	0	0	-	0	519 ± 4	0.78	516 ± 4	0.32	1.444	5.995
	12.1	12.1 ± 0.4	1.3							
	48.5	48.5 ± 0.6	0.77							
3 (x3)	0	0	-	0	700 ± 9	1.0	703 ± 18	1.0	0.423	1.019
	12.1	12.1 ± 0.4	1.3							
	24.3	24.3 ± 0.6	1.9							

^1 ^*t*_crit_ = 2.45 at α = 0.05 and *f* = 6. ^2^ *F*_crit_ = 6.94 at *α* = 0.05 and *f*_1_ = 4, *f*_2_ = 2.

## Data Availability

The data presented in this study are available in the electronic Supplementary Materials.

## Supplementary Materials

The following are available online at https://www.mdpi.com/article/10.3390/s22010288/s1, Figure S1: Effect of pulse parameters on the oxidation potentials (a,b) and oxidation currents (c,d) of 5.0 µM mixture of ferulic acid (a,c) and vanillin (b,d) on polyBCP/f-SWCNTs/GCE in BRB, pH 2.0, Figure S2: (a) Baseline-corrected DPV of 10 and 2.5 µM gallic acid on polyBCP/f-SWCNTs/GCE in BRB, pH 2.0. (b) Baseline-corrected DPV of 1.0 and 0.25 µM vanillic acid on polyBCP/f-SWCNTs/GCE in BRB, pH 2.0; Δ*E*_pulse_ = 75 mV; *t*_pulse_ = 25 ms; υ = 10 mV s^−1^, Table S1: Recovery of ferulic acid and vanillin in *Vanilla planifolia* extract (*n* = 5; *p* = 0.95).
