# Supplementary material for: Simultaneous Determination of Ferulic Acid and Vanillin in Vanilla Extracts Using Voltammetric Sensor Based on Electropolymerized Bromocresol Purple
Source: Sensors (Basel). 2021 Dec 31;22(1):288. doi: 10.3390/s22010288 (PMC8749893; doi:10.3390/s22010288)
Supplement: Supplementary file 1 [file sensors-22-00288-s001.zip › sensors-1506110-supplementary.pdf]

Supplementary Materials

Simultaneous Determination of Ferulic Acid and Vanillin in Vanilla Extracts Using Voltammetric Sensor Based on the Electropolymerized Bromocresol Purple

Guzel Ziyatdinova, Anastasiya Zhupanova and Rustam Davletshin

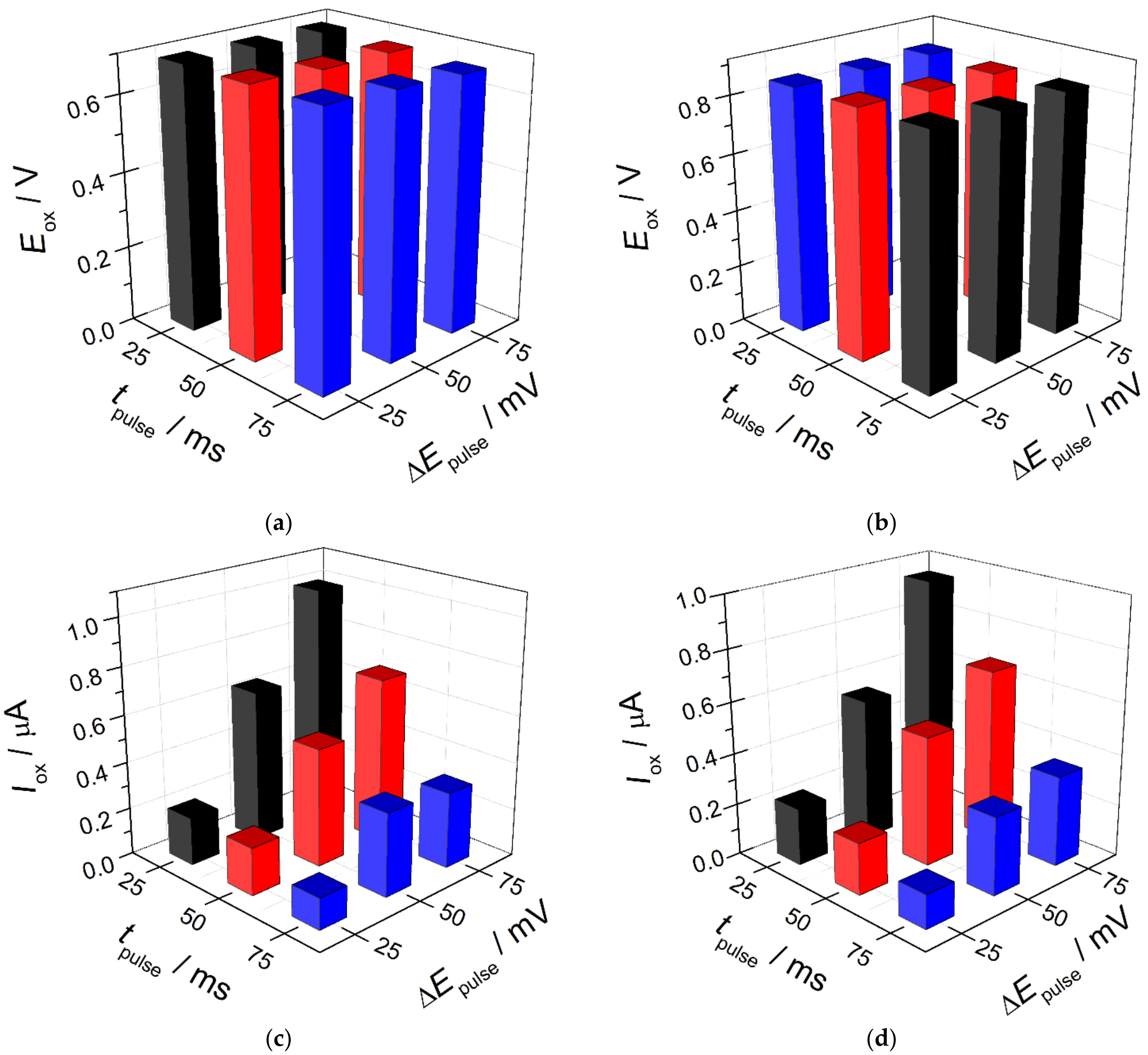

Figure S1. Effect of pulse parameters on the oxidation potentials (**a** and **b**) and oxidation currents (**c** and **d**) of 5.0 µM mixture of ferulic acid (**a, c**) and vanillin (**b**) at the polyBCP/f-SWCNTs/GCE in BRB pH 2.0.

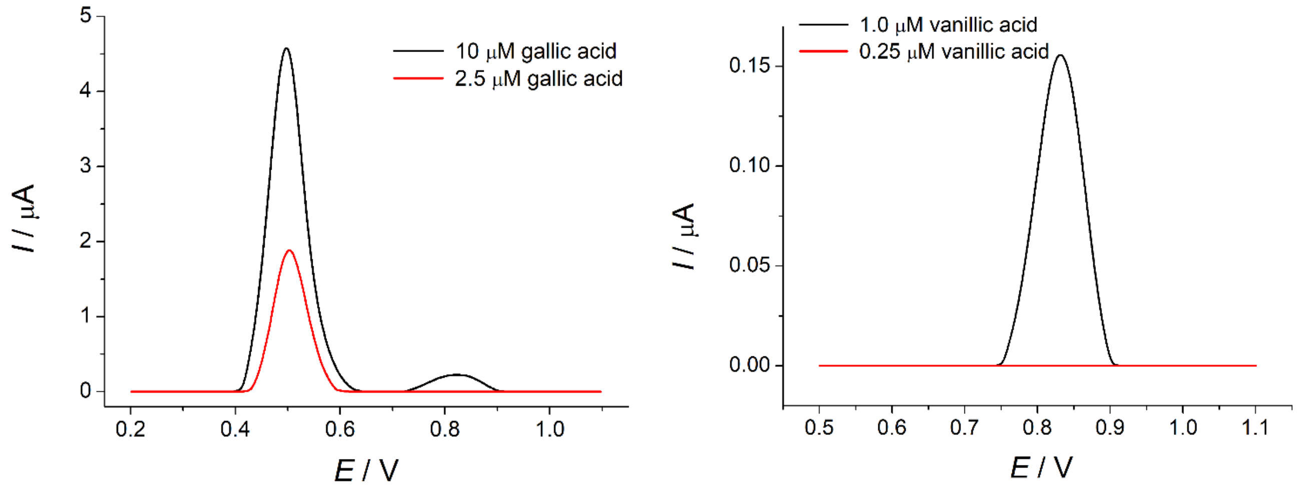

(**a**) (**b**)

Figure S2. (**a**) Baseline-corrected DPV of 10 and 2.5 μM gallic acid at the polyBCP/f-SWCNTs/GCE in BRB pH 2.0; (**b**) Baseline-corrected DPV of 1.0 and 0.25 μM vanillic acid at the polyBCP/f-SWCNTs/GCE in BRB pH 2.0. ΔE_{pulse} = 75 mV, t_{pulse} = 25 ms, υ = 10 mV s^{-1}.

Table S1. Recovery of ferulic acid and vanillin in *Vanilla planifolia* extract (*n* = 5; *P* = 0.95).

Analyte	Spiked/μM	Found/μM	RSD/%	*R*/%
	0	0	-	-
Ferulic acid	0.50	0.50 ± 0.01	2.0	100
	1.0	1.00 ± 0.03	2.8	100
	0	1.67 ± 0.06	1.5	-
Vanillin	0.83	2.49 ± 0.04	1.2	99.6
	1.67	3.34 ± 0.09	1.1	100
